# Tuberculosis of the prostate and urethra: A review

**DOI:** 10.4103/0970-1591.42623

**Published:** 2008

**Authors:** Nitin Gupta, A. K. Mandal, S. K. Singh

**Affiliations:** Department of Urology, PGIMER (Post Graduate Institute of Medical Education and Research), Chandigarh, India

**Keywords:** Genitourinary, granulomatous, infection, prostate, rare, tuberculosis, urethra

## Abstract

Genitourinary tuberculosis contributes to 10-14% of extrapulmonary tuberculosis and is a major health problem in India. Prostate tuberculosis is uncommon and is usually found incidentally following transurethral resection. The most common mode of involvement is hematogenous, though descending infection and direct intracanalicular extension is known. Predisposing factors include prior tubercular infection, immuno-compromised status, previous BCG therapy. The presentation is diffuse caseating epitheloid cell granulomas, which can be confirmed by prostate biopsy. Urine PCR has good sensitivity (95.5%) and specificity ( 98.12%) in diagnosis. Imaging techniques like TRUS and CT/MRI also allow good visualization of the lesion and its extension. Urethral tuberculosis is very rare and is usually secondary to upper tract or genital tuberculosis. The presentation may be acute urethritis or chronic stricture or fistulae. The treatment of choice is chemotherapy with 3-4 anti tubercular drugs for initial 6-12 weeks and later 2 drugs for additional 3-6 months. Surgery is usually reserved for cases where chemotherapy fails and is done after 4-6 weeks of ATT. With a high index of suspicion it may be possible to diagnose a larger number of cases of prostatic and urethral tuberculosis especially in this country where tuberculosis is almost endemic.

## INTRODUCTION

Tuberculosis (TB) is a major public health problem in developing countries. Worldwide also, TB continues to be an important clinical problem, mainly because of its nonspecific clinical presentation and variable radiographic appearance. Genitourinary tuberculosis (GUTB) has been reported to contribute 10-14% of the extrapulmonary tuberculosis with involvement of any part from kidney to urethra.[1] With an estimated over 10 million sufferers of TB, GUTB constitutes a major urological problem in India. It is a form of secondary TB, the symptoms and signs of which are often vague and insidious. A high index of suspicion helps in early diagnosis.

## PROSTATE TUBERCULOSIS

Prostate TB is much less common than renal, vesiculo-seminal and epididymal TB. Thus many urologists are unfamiliar with the diagnosis and management of prostatic TB with many cases found incidentally following transurethral resection.[2]

The possible modes of involvement include a descending infection from the urinary organs, direct intracanalicular extension from a neighboring tuberculous focus in the genital tract or a hematogenous spread. On the basis of clinical observations and animal experiments, Sporer *et al.*,[3] suggested that TB of the prostate is almost always the result of one or perhaps successive hematogenous seedings. Direct extension may occur; however, descending infection of the prostate has never been encountered.[3] It is well established that the predisposing factors associated with the development of TB include prolonged steroid use, immunosuppressive therapy, diseases that impair cell-mediated immunity, and diseases with poor immune mechanisms.[2] Extrapulmonary TB has been reported to be steadily increasing in patients with acquired immunodeficiency syndrome (AIDS).[4]

**Figure 1:**
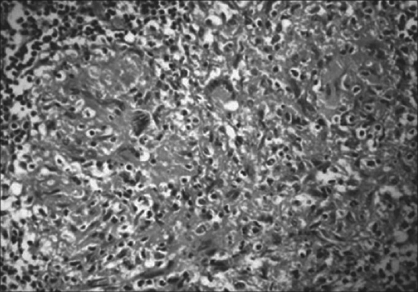
Tuberculous prostatis showing multiple caseating epitheloid granulomas with Langhan's giant cells (H and E, ×100)

Tuberculosis of the prostate gland presents with diffuse caseating epithelioid cell granulomas which are not confined to the periglandular/periductal region, as seen in cases of nonspecific granulomatous prostatitis (NSGnP). Other infectious agents, such as *Treponema pallidum*, [Figure 1] viruses[5] and various fungi,[6] are rare causes of granulomatous prostatitis. Histochemical stains like PAS, Gomori's stain and Ziehl-Nielson stain are helpful in confirming infectious etiology.

In most cases the cause of granulomatous prostatitis (GnP) is unknown,[7] but GnP can occur after various predisposing/precipitating events, e.g. urinary tract infections(71%)[8], transurethral resection of prostate/ open prostatectomy[9] and needle biopsy.[10] Recently, a higher incidence of GnP was found in patients who had been treated with intravesical bacille Calmette-Gue'rin.[11–13] Nonspecific granulomatous prostatitis is usually an incidental finding, with an incidence of < 3.4% in unselected series of patients;[14] it is detected in 0.44% of routine prostatectomy specimens and in 0.29[9] to 3.3%[15] of needle prostate biopsies. It is important to differentiate NSGnP from specific granulomatous prostatitis, as this type is a self-limiting benign condition, while the latter requires specific treatment.[16]

## URETHRAL TUBERCULOSIS

Urethral TB is very rare despite the constant exposure of the urethra to the infected urine. Most often it has been reported to occur in association with upper tract involvement or female genital involvement.[17] Isolated urethral involvement is extremely uncommon. Tuberculosis of the urethra is usually due to the spread from another focus in the genitourinary tract, the prostate being the common source.[18] The exact incidence of urethral involvement in TB is not known. Female urethral TB is probably still rarer as compared to male urethral involvement with the spread from uterus and cervix being the important source in them. Symes and Blandy[18] have reported five cases of urethral TB out of 112 male patients having urethral stricture. Ross[19] reported nine cases of urethral involvement out of 469 patients with GUTB. Indudhara *et al.*,[20] have reported their experience with two cases; one was a male patient who presented with urethral stricture and perineal fistulae, wherein histopathology of the excised scar tissue revealed TB. The other one was a female who presented with a urethral caruncle which was excised and histopathology revealed features of TB.

## DIAGNOSIS

Diagnosing prostatic TB can be challenging. The most critical step in attempting to elucidate a diagnosis of genitourinary TB is from the patient's clinical history.[21] Prior TB infection as a child, immunocompromised states, such as human immunodeficiency virus/acquired immunodeficiency syndrome, immunosuppression with organ transplantation, travel to endemic areas, and immigration are important considerations when obtaining the medical history.[4] The latency between pulmonary TB and manifestations seen in the genitourinary tract can be lengthy, with some reports showing a period of 30 years before the disease making an appearance. Tubercular prostatitis should also be suspected in patients with lower urinary tract symptoms and prostatic tenderness or nodularity after undergoing bacille Calmette-Guérin therapy for bladder cancer.[22]

- **TRUS:** The tuberculous lesions are typically located in the peripheral part of the posterior and lateral lobes of the prostate.[23] The TRUS findings of diffuse hypoechoic lesions within the peripheral zone of the prostate makes it frequently difficult to differentiate between prostatic TB and adenocarcinoma of the prostate. The diagnosis can only be confirmed by prostate biopsy. However, TRUS is one of the tools useful for the diagnosis of prostatic abscess, TRUS allows excellent visualization of the prostatic anatomy and the relationship of the abscess to the prostatic lobes, and permits appropriate transurethral unroofing.[24]
- **Computed Tomography/ Magnetic Resonance Imaging scan:** CT findings have also been shown to be consistent with the findings of previous reports that the round areas of decreased attenuation within the prostate suggest a tuberculous abscess.[25] CT can also demonstrate the extension of the abscess and the involvement of the adjacent organ.[24] Prostatic tuberculous cavities or abscesses may discharge into the surrounding tissues, forming sinuses or fistulae to the perineum or rectum and eventually resulting in a watering-can perineum. These changes are demonstrated best on MRI scans.
- **Immunological tests:** Positive Siebert purified protein derivative of tuberculin test (PPD) supports TB infection, but a negative test does not rule it out.[26] A definitive diagnosis is made by positive cultures, Ziehl-Nielsen staining, and/or histological examination [Figure 1].[4,26] However, staining has a low sensitivity (52.7% in one study), especially in nonpulmonary TB, and cultures require up to eight weeks for maximal sensitivity to be reached.[4,26,27]
- **Molecular diagnosis:** Polymerase chain reaction (PCR) is becoming a useful clinical diagnostic tool because of its rapid detection and high sensitivity and specificity. Moussa *et al.*,[27] reported the sensitivity and specificity of PCR of urine to be 95.59% and 98.12%, respectively. However, one of the disadvantages of PCR is its inability to detect whether the TB infection is biologically active or in its latent phase.[4] Most investigators suggest using PCR in combination with cultures and Ziehl-Nielsen staining when making a diagnosis and developing a treatment plan.[4,26,27]

Urethral TB may present as acute urethritis with urethral discharge and associated affection of the prostate, seminal vesicles, and other parts of the urinary tract or the female genital tract or as the chronic variant with stricture formation.[28] Confirmation of the diagnosis in acute variant is probably not difficult while in the later it is difficult. The chronic form may present in bizarre and unexpected forms with fistulae of unusual types and difficult strictures not responding to conventional urethroplasty. Biopsy of the scar tissue may suggest tubercular affection.

## TREATMENT

Since 1982, the American Thoracic Society and the Centers for Disease Control have recommended a nine-month course of isoniazid and rifampicin for the routine treatment of TB in the United States.[29] However, a shorter course of four or six months of chemotherapy has been recommended for the treatment of tuberculosis.[30] Gow *et al.*, suggested that it was not necessary to extend chemotherapy beyond four months, except in unusual circumstances.[31,32]

The treatment of choice is chemotherapy using three to four anti-TB drugs for up to six to nine months.[4,14,26,33,34] Isoniazid, rifampicin, and pyrazinamide, with or without ethambutol, are normally used initially for six to 12 weeks. After the six to 12-week course, isoniazid and rifampin are used for an additional three to six months. However, resistance is developing, particularly to rifampicin, isoniazid, and streptomycin.[4,26,33]

In spite of effective antitubercular agents, surgery remains an important part of treatment plan of GUTB, especially as it may have been present for years before diagnosis and as abscesses from between prostate and urethra. Additionally, extensive prostatic and urethral involvement may be resistant to chemotherapy.[14,16,33,34] Buchholz *et al.*,[26] reported that 52% of surgical specimens showed florid TB despite a previous nine-month course of anti-TB chemotherapy. Also, patient compliance and follow-up are important factors that affect and can complicate the success of anti-TB chemotherapy.[26] The role of the DOTS Program which has been successfully tried in pulmonary TB is being evaluated in GUTB to improve patient compliance. Unless advanced disease is present, such as abscess formation, chemotherapy is recommended first, with surgery used as a second- line intervention when chemotherapy fails.

Lee *et al.*,[35] reported results of a triple-drug regimen of six months for prostatic TB. After a median follow-up of 3.4 years (range one to nine), no relapse had been observed. These findings suggest that a short-term regimen may be sufficient for prostatic TB.

However, a four-month regimen or even the standard six or nine-month course of chemotherapy may not be suitable for patients infected with HIV, because relapses have been reported.[36–38] Infection with HIV increases the risk of TB and is thought to decrease the effectiveness of antituberculosis treatment.[39] Unlike other patients, the tuberculous infection of the prostate in patients with acquired immunodeficiency syndrome were mostly prostatic abscess.[24,40,41] Moreover, the outbreaks of multidrug-resistant tuberculosis (MDR-TB) in persons with or without HIV infection are associated with higher mortality.[42,43] Thus, a different strategy should be applied in patients with MDR-TB or HIV or other severely immunocompromised status. Histological follow-up is a good method for monitoring the efficacy of treatment. Periodic transrectal biopsies to evaluate the efficacy of antituberculosis treatment have been successfully used for follow-up.[35] Consistent with this notion is the report indicating that after an appropriate course of chemotherapy, residual induration can be biopsied percutaneously.[44] Fine needle aspiration cytology is a suitable alternative for the diagnosis and follow-up of prostatic TB.[45,46]

Antitubercular therapy (ATT) is highly effective, and in most cases curable. Surgical intervention is required only in a minority of cases.

Patients diagnosed to have urethral TB should be given ATT for at least six weeks before any surgical intervention in order to prevent reactivation of a latent focus in the dense scar tissue. The strictures can be treated on conventional lines similar to any urethral stricture. However, urethroplasty may be preferred in the presence of dense fibrous scar involving the urethra and periurethral tissue. The timing of urethroplasty is not clearly defined. However, all reconstructive procedures on the genitourinary tract are done after an initial four to six weeks of ATT.

## CONCLUSION

Prostatic and urethral involvement by *Mycobacterium tuberculosis* is rather uncommon. Its rarity is difficult to understand in view of the almost constant exposure of the urethra to the infected urine. A complete understanding of the cause and optimal treatment of prostatic TB and periodic histological follow-up to determine the efficacy of chemotherapy are especially beneficial for patients with MDR-TB or HIV or other severely immunocompromised conditions.

The diagnosis of the tuberculous etiology of the stricture urethra is not easily proven and this makes one think that urethral tuberculosis may be more common than one observes from a study of the literature. With a high index of suspicion and availability of sophisticated and reliable tests for molecular diagnosis like PCR, RT-PCR etc. it may be possible to diagnose a larger number of cases of urethral and prostatic tuberculosis especially in this country where tuberculosis is almost endemic.
